# Impact of *SLCO1B1* Polymorphism and Vitamin D Status on Statin Efficacy and Tolerability in Postmenopausal Women

**DOI:** 10.3390/biomedicines14010113

**Published:** 2026-01-06

**Authors:** Romana Marušić, Dunja Šojat, Tatjana Bačun, Nenad Nešković, Željko Debeljak, Mirna Glegj, Melita Vukšić Polić, Saška Marczi

**Affiliations:** 1 Faculty of Medicine Osijek, Josip Juraj Strossmayer University of Osijek, 31000 Osijek, Croatia; romana.marusic55@gmail.com; 2 National Memorial Hospital “Dr. Juraj Njavro” Vukovar, 32000 Vukovar, Croatia; 3 Health Center of Osijek-Baranja County, 31000 Osijek, Croatia; dunja.sojat@gmail.com; 4 Department of Family Medicine, Faculty of Medicine, Josip Juraj Strossmayer University Osijek, 3100 Osijek, Croatia; 5 Department of Endocrinology, Internal Medicine Clinic, University Hospital Centre Osijek, 31000 Osijek, Croatia; 6 Department of Internal Medicine and History of Medicine, Josip Juraj Strossmayer University Osijek, 31000 Osijek, Croatia; 7 Department of Clinical Medicine, Faculty of Dental Medicine and Health Osijek, Josip Juraj Strossmayer University Osijek, 31000 Osijek, Croatia; 8 International Medical Center Priora, 31431 Čepin, Croatia; nneskov@gmail.com; 9 Department of Anesthesiology and Intensive Care Medicine, Faculty of Medicine, Josip Juraj Strossmayer University Osijek, 31000 Osijek, Croatia; 10 Clinical Institute of Laboratory Diagnostics, University Hospital Centre Osijek, 31000 Osijek, Croatia; zeljko.debeljak@gmail.com; 11 Department of Pharmacology, Faculty of Medicine, Josip Juraj Strossmayer University of Osijek, 31000 Osijek, Croatia; 12 Department of HLA Typing and Genomic Diagnostics, Clinical Institute of Transfusion Medicine, University Hospital Centre Osijek, 31000 Osijek, Croatia; glegj.mirna@kbco.hr (M.G.); saska.marczi@kbco.hr (S.M.); 13 Department of Dermatology and Venereology, University Hospital Centre Osijek, 31000 Osijek, Croatia; mvuksic@mefos.hr; 14 Department of Dermatology, Faculty of Medicine, Josip Juraj Strossmayer University Osijek, 31000 Osijek, Croatia; 15 Department of Medical Chemistry, Biochemistry and Clinical Chemistry, Josip Juraj Strossmayer University Osijek, 31000 Osijek, Croatia

**Keywords:** hydroxymethylglutaryl-CoA reductase inhibitors, *SLCO1B1* gene, vitamin D deficiency, postmenopause, pharmacogenetics

## Abstract

**Background**: Interindividual differences in statin efficacy and tolerability are partly determined by genetic and metabolic factors. The *SLCO1B1* c.521T>C polymorphism affects hepatic statin transport, while vitamin D deficiency may influence lipid metabolism and muscular tolerance. This study aimed to assess the impact of *SLCO1B1* genotype and vitamin D status on lipid-lowering response and adverse events in postmenopausal women treated with atorvastatin or rosuvastatin. **Methods**: A total of 145 Croatian postmenopausal women were prospectively followed for 16 weeks. Participants received atorvastatin or rosuvastatin with dose titration to achieve low-density lipoprotein cholesterol (LDL-C) targets. Serum lipids, liver enzymes, and creatine kinase were monitored monthly. Serum levels of 25-hydroxyvitamin D were quantified by LC–MS/MS, while *SLCO1B1* c.521T>C genotyping was performed using real-time PCR. **Results**: Rosuvastatin achieved a higher LDL-C target attainment rate compared with atorvastatin (81.1% vs. 67.6%, *p* = 0.02). The *SLCO1B1* genotype was not associated with lipid response but was significantly associated with adverse effects. In multivariable regression analysis, patients with the T/C genotype had a significantly higher risk of developing adverse effects compared with those with the T/T genotype (OR 7.4, 95% Cl 2.1–26.7, *p* = 0.002). Vitamin D status showed no significant association with lipid outcomes or adverse events, although participants with severe deficiency exhibited a weaker LDL-C response. **Conclusions**: Rosuvastatin demonstrated superior lipid-lowering efficacy and tolerability compared with atorvastatin in postmenopausal women. The *SLCO1B1* c.521T>C variant primarily affected safety rather than efficacy, while severe vitamin D deficiency might contribute to diminished statin response. Integrating pharmacogenetic and endocrine profiling could enhance individualized statin therapy and cardiovascular prevention in women.

## 1. Introduction

Cardiovascular diseases (CVDs) remain the leading cause of morbidity and mortality worldwide. According to the World Health Organization, approximately 19.8 million people died from CVDs in 2022, accounting for around 32% of all global deaths [1]. The development of cardiovascular disease is a multifactorial process influenced by genetic, environmental, and metabolic factors.

Atherosclerosis develops years before clinical manifestation and is accelerated by both non-modifiable (age, sex, genetic predisposition) and modifiable risk factors, including arterial hypertension, dyslipidemia, diabetes mellitus, smoking, overweight and obesity, sedentary lifestyle, unhealthy diet, and excessive alcohol intake [2,3,4].

Statins are first-line lipid-lowering agents that effectively reduce low-density lipoprotein cholesterol (LDL-C) and significantly lower the risk of atherosclerotic cardiovascular disease (ASCVD). Their broad clinical use and robust evidence base have made them one of the most prescribed drug classes globally [5].

Lowering LDL-C is a cornerstone of cardiovascular prevention. A 1 mmol/L reduction in LDL-C is associated with an approximately 22% decrease in the five-year risk of major cardiovascular events, while statin therapy typically reduces LDL-C by 20–50% and translates into a ~25% reduction in myocardial infarction risk and a ~15% reduction in stroke risk [6,7].

Despite their proven efficacy, substantial interindividual variability in both lipid-lowering response and the development of adverse effects persists, limiting adherence and therapeutic outcomes. These differences are influenced by genetic factors, comorbidities, hormonal status, and environmental determinants.

Among genetic determinants, the *SLCO1B1* gene, encoding the influx transporter organic anion-transporting polypeptide 1B1 (OATP1B1), plays a key role in the hepatic uptake of several statins, including atorvastatin and simvastatin, and to a lesser extent rosuvastatin [7,8,9]. The most extensively investigated is single-nucleotide polymorphism rs4149056 (c.521T>C, p.Val174Ala), a missense variant that reduces OATP1B1 activity. Consequently, carriers of the C allele (T/C or C/C genotypes) exhibit higher systemic exposure to statins, predisposing them to statin associated muscle symptoms (SAMS) [8,10]. These pharmacokinetic alterations may also affect lipid-lowering efficacy, as increased plasma concentrations do not necessarily correspond to enhanced hepatic LDL receptor expression due to reduced intracellular statin transport [9].

Apart from genetic predisposition, vitamin D deficiency has been implicated as a modifiable factor influencing both statin intolerance and treatment response. Vitamin D plays an essential role in skeletal muscle maintenance, mitochondrial function, and inflammatory regulation [11,12]. Low 25-hydroxyvitamin D levels have been associated with a higher incidence of myalgia and SAMS, possibly through impaired calcium homeostasis and altered muscle energy metabolism [13]. Furthermore, vitamin D modulates the expression of *CYP3A4* gene, a key cytochrome P450 enzyme responsible for atorvastatin metabolism [13,14,15].

Postmenopausal women represent a particularly vulnerable population. Estrogen deficiency during menopause promotes unfavorable lipid profile changes—elevated total and LDL cholesterol with reduced high-density lipoprotein cholesterol (HDL-C)—as well as decreased vitamin D synthesis and bone mass [16]. These patients frequently require statin therapy but may be more prone to statin-related muscle and metabolic adverse effects. Despite their high prevalence of both dyslipidemia and vitamin D deficiency, data on the interaction between *SLCO1B1* polymorphism, vitamin D status, statin efficacy, and intolerance in postmenopausal women remain limited.

Previous studies suggest that rosuvastatin may be better tolerated than atorvastatin in carriers of the *SLCO1B1* C allele, likely due to its hydrophilic properties and reduced reliance on OATP1B1-mediated hepatic uptake [7,17].

However, published data are inconsistent, and it remains unclear how *SLCO1B1* genotype and vitamin D status jointly influence statin tolerability. Clarifying these interactions may support a more individualized approach to lipid-lowering therapy.

The present study aimed to evaluate the effects of *SLCO1B1* genetic polymorphism and vitamin D concentration on (1) the statin dose required to achieve LDL-C targets, (2) the lipid-lowering response over time, and (3) the occurrence of statin-associated adverse effects, including gastrointestinal symptoms, muscle-related symptoms, and biochemical abnormalities, specifically elevated hepatic transaminases and creatine kinase (CK), in postmenopausal women treated with atorvastatin or rosuvastatin. Understanding these associations may provide valuable insights into the mechanisms underlying interindividual statin variability and support a more personalized approach to cardiovascular risk reduction.

## 2. Materials and Methods

### 2.1. Study Design and Setting

This prospective study was conducted across more than 30 family medicine practices of the Osijek-Baranja County Health Center (Croatia). Participants were followed monthly for 16 weeks (baseline and follow-up visits at months 1–4). The study protocol was approved by the institutional ethics committees (Ethics Committee KLASA: 602-04/21-08/02, URBROJ: 2158-61-07-21-176), and all participants provided written informed consent prior to enrollment. Participants were informed about the purpose of the research, their rights, and the voluntary nature of participation, including the option to withdraw at any time. They had opportunity to ask questions before providing informed consent. All data were collected anonymously and handled in accordance with ethical guidelines.

### 2.2. Participants

Eligible participants were postmenopausal women (45–70 years; ≥12 months of amenorrhea) with an indication for statin therapy according to current European Society of Cardiology/European Atherosclerosis Society (ESC/EAS) guidelines [18]. Exclusion criteria included iatrogenic menopause, active malignancy, hormone replacement therapy, alcohol abuse, diabetes mellitus, uncontrolled hypertension requiring major therapy changes, active liver disease or persistent transaminase elevation (>3× ULN), renal insufficiency, untreated thyroid dysfunction, prior statin intolerance or allergy, current use of vitamin D or other lipid-modifying drugs (fibrates, ezetimibe, proprotein convertase subtilisin/kexin type 9 (PCSK9) inhibitors), and concomitant use of strong *CYP3A4* inhibitors.

Participants were assigned to atorvastatin or rosuvastatin treatment groups using block randomization to balance group sizes. The initial statin intensity (moderate or high) was determined based on the required LDL-C reduction, and doses were up-titrated at each monthly visit until LDL-C targets were achieved or the maximally tolerated dose was reached (atorvastatin up to 80 mg, rosuvastatin up to 40 mg).

### 2.3. Procedures and Measurements

At the baseline visit, anthropometric parameters (weight, height, body mass index (BMI), and waist-to-hip ratio), blood pressure, smoking habits, physical activity, and alcohol intake were recorded. Laboratory analyses were performed at every monthly visit, including fasting venous blood sampling for total cholesterol (TC), LDL-C, HDL-C, triglycerides (TG), glucose, and safety parameters (alanine aminotransferase (ALT), aspartate aminotransferase (AST), gamma-glutamyl transferase (GGT), CK), determined by standard enzymatic methods on automated analyzers, specifically using the AU700 analyzer (Beckman Coulter, Indianapolis, IN, USA).

Serum concentrations of 25-hydroxyvitamin D3 [25(OH)D3] were measured by liquid chromatography–tandem mass spectrometry (LC–MS/MS; LCMS-8050, Shimadzu, Kyoto, Japan). Vitamin D status was classified according to serum concentration as follows: Severe Deficiency was defined as 0–10 ng/mL (0–25 nmol/L), Deficiency as >10–20 ng/mL (>25–50 nmol/L), Suboptimal/Insufficiency as >20–30 ng/mL (>50–75 nmol/L), and Optimal as >30–50 ng/mL (>75–125 nmol/L). Vitamin D was determined after achieving LDL-C targets or, if targets were not met, at the final 16-week visit.

Treatment adherence was assessed at each visit by direct patient interview and pill count. Adherence ≥80% of prescribed doses was considered acceptable and required for inclusion in the final analysis.

SAMS were defined as muscle pain or weakness with or without creatine kinase elevation, temporally related to statin use and resolving upon dose reduction or discontinuation. The presence of muscle symptoms was systematically monitored through structured patient interviews at each follow-up visit. Causality was assessed using the Naranjo algorithm.

### 2.4. Genotyping

Genomic DNA was isolated from EDTA whole blood. The *SLCO1B1* c.521T>C (rs4149056) polymorphism was genotyped using LightSNiP SLCO1B1 T521C Kit (TIB Molbiol, Berlin, Germany) and LightCycler FastStart DNA Master HybProbe (Roche Diagnostics, Mannheim, Germany) with melting-curve analysis on a LightCycler 480 II platform (Roche Diagnostics, Rotkreuz, Switzerland) following the manufacturer’s protocol.

### 2.5. Outcomes

Primary outcomes were the effects of *SLCO1B1* genetic polymorphism and vitamin D concentration on the following:

Achievement of LDL-C target values;

Required statin dose;

Incidence of statin-associated adverse effects.

Secondary outcomes included longitudinal changes in lipid parameters (LDL-C, HDL-C, TG, TC) by statin type, dose, and genotype.

### 2.6. Statistical Analysis

Continuous variables were tested for normality (Shapiro–Wilk) and expressed as median (interquartile range, IQR), while categorical variables were presented as counts and percentages. Between-group comparisons used the Mann–Whitney U test, and categorical data were compared by χ^2^ test. Longitudinal changes were analyzed using Friedman test with post hoc Conover.

To identify independent predictors of achieving the therapeutic target and the occurrence of adverse events, multivariable logistic regression analysis was performed using a stepwise forward selection procedure based on the likelihood ratio. Variables that demonstrated a potential association in univariable analyses (*p* < 0.10) or were considered clinically relevant were included in the multivariable model. A *p*-value < 0.05 was considered statistically significant, and all *p*-values were two-sided.

Analyses were performed using MedCalc v20.010 and IBM SPSS Statistics v23.

## 3. Results

### 3.1. Participant Flow and Study Population

**Figure 1:**
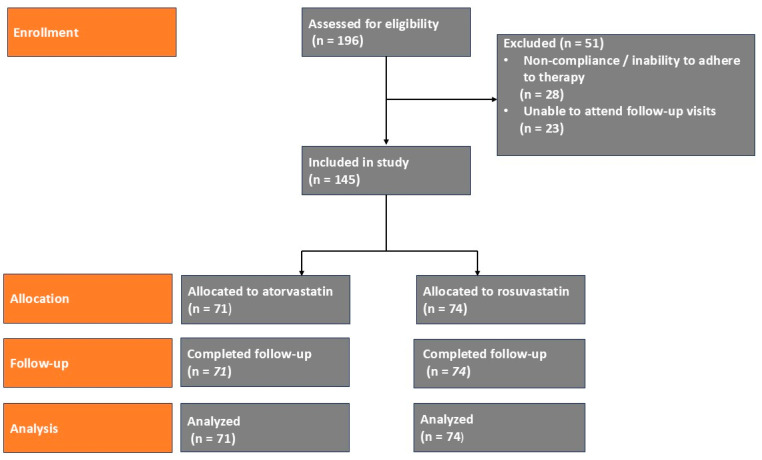
CONSORT-style flow diagram of participant enrollment, allocation, follow-up, and analysis.

Participant flow through the study is summarized in a CONSORT-style flow diagram (Figure 1). A total of 196 individuals were assessed for eligibility; 51 were excluded due to non-compliance with therapy or inability to attend follow-up visits, leaving 145 participants included in the final analysis.

### 3.2. Baseline Characteristics of Participants

We enrolled 145 postmenopausal women with a median age of 60 years (IQR 55–64) and menopause onset at 50 years (48–52). Median BMI was 27.0 kg/m^2^ (24.1–30.9). Most participants were non-smokers or former smokers (66.2%), while roughly one third reported current smoking. More than half consumed alcohol at least occasionally in the previous year, though daily intake was uncommon (3.4%). Median total daily moderate-to-vigorous activity was 120 min (75–200), with a median sedentary time of 240 min (120–360). By Systematic Coronary Risk Evaluation 2 for Older Persons (SCORE2-OP) categories, 29.0% were at low-to-moderate, 37.2% at high, and 33.8% at very-high cardiovascular risk. Detailed demographic, anthropometric, lifestyle and activity data are presented in Table 1.

### 3.3. Statin Treatment Groups and SLCO1B1 Genotype Distribution

Of the 145 participants, 71 (49%) received atorvastatin and 74 (51%) rosuvastatin. Baseline demographic and laboratory characteristics were comparable between treatment groups, except for triglyceride levels, which were higher in the rosuvastatin group (median 1.4 [1.0–2.0] mmol/L) than in the atorvastatin group (1.1 [0.9–1.7] mmol/L; Mann–Whitney test, *p* = 0.03).

Genotyping of the *SLCO1B1* c.521T>C polymorphism identified T/T in 62.8%, T/C in 32.4%, and C/C in 4.8% of participants. Baseline clinical and biochemical characteristics were similar across genotype groups, except for fasting glucose levels, which were slightly higher in T/T carriers (median 5.1 [4.6–5.4] mmol/L) compared with T/C heterozygotes (4.8 [4.4–5.1] mmol/L; Kruskal–Wallis test with post hoc Dunn correction, P_holm = 0.02).

### 3.4. Statin Dosage and Achievement of LDL-C Targets

At baseline, 67.6% of participants received high-intensity statin therapy (atorvastatin ≥40 mg or rosuvastatin ≥20 mg), with no significant difference in dose distribution between treatment groups. Dose intensity remained stable throughout the 16-week follow-up (Table 2).

Median LDL-C decreased from 3.8 mmol/L (IQR 3.2–4.4) to 2.2 mmol/L (1.9–2.7), representing a relative reduction of 42% (*p* < 0.001). LDL-C targets, defined according to the 2021 ESC/EAS dyslipidemia guidelines, were achieved in 74.5% of participants. Goal attainment was significantly higher in the rosuvastatin group (81.1%) compared with the atorvastatin group (67.6%, *p* = 0.02) (Table 3).

### 3.5. Longitudinal Changes in Lipid Parameters

(a) According to statin type

**Figure 2:**
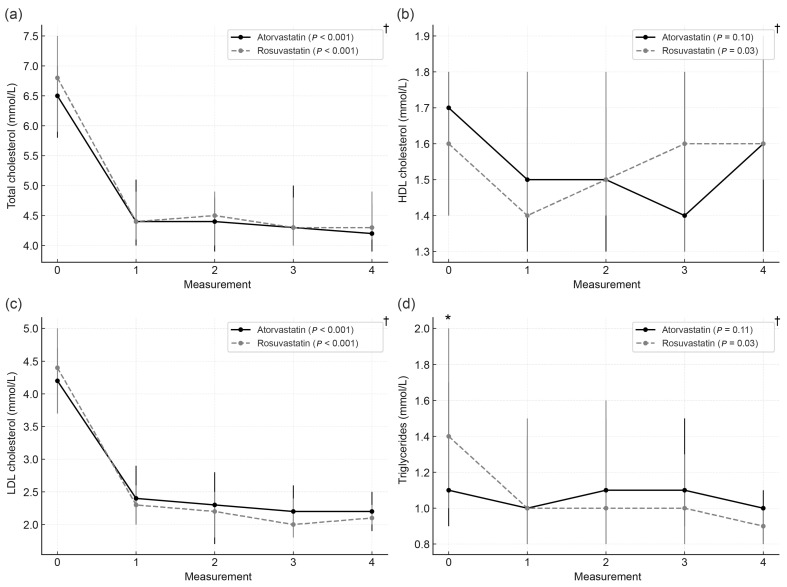
Longitudinal changes in lipid parameters according to statin type. (**a**) Total cholesterol, (**b**) HDL cholesterol, (**c**) LDL cholesterol, and (**d**) triglycerides during follow-up. Data are presented as medians with interquartile ranges. Measurement 0: baseline visit; 1–4: follow-up visits. * Mann–Whitney test *p* < 0.05; † Friedman test.

Across the total sample, a ≥50% reduction in LDL-C from baseline was achieved in 94 participants (64.8%), including 42.6% of atorvastatin users and 57.4% of rosuvastatin users (*p* = 0.09). During follow-up, the rosuvastatin group showed a significant reduction in all lipid fractions, whereas in the atorvastatin group, a decline was observed only in total and LDL cholesterol. Median lipid concentrations at baseline and follow-up visits by statin type are shown in Figure 2.

(b) According to *SLCO1B1* c.521T>C genotype

No statistically significant differences in total cholesterol, HDL-C, LDL-C, or triglyceride levels were observed between *SLCO1B1* genotypes at any time point. Likewise, the proportion of participants achieving target LDL-C levels did not differ significantly across genotypes during follow-up.

Within the *SLCO1B1* T/T subgroup, total cholesterol, HDL-C, and LDL-C levels significantly decreased after statin initiation, while triglycerides remained unchanged. Among T/C carriers, total cholesterol, LDL-C, and triglycerides decreased significantly, with no relevant change in HDL-C. In contrast, participants with the C/C genotype showed no significant change in any lipid fraction during follow-up.

**Figure 3:**
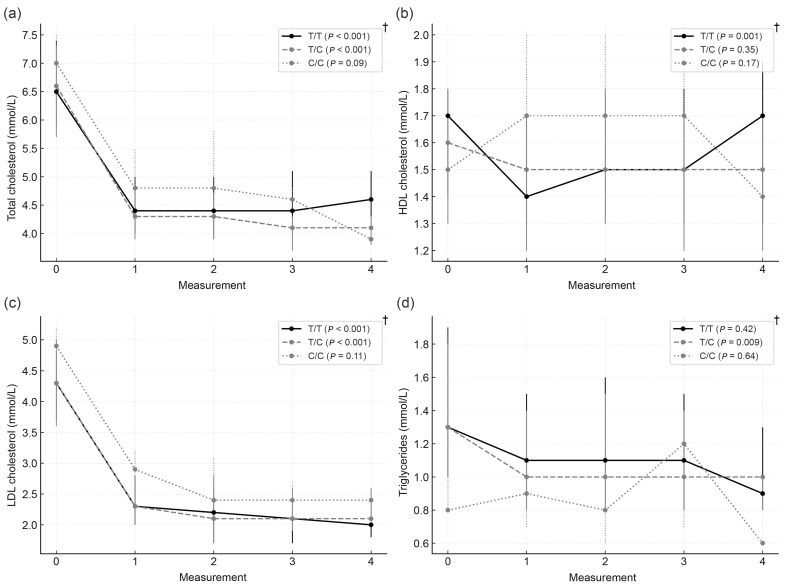
Longitudinal changes in lipid parameters according to SLCO1B1 genotype. (**a**) Total cholesterol, (**b**) HDL cholesterol, (**c**) LDL cholesterol, and (**d**) triglycerides during follow-up. Data are presented as medians with interquartile ranges. Measurement 0: baseline visit; 1–4: follow-up visits. † Friedman test.

Median concentrations of lipid parameters across four follow-up visits according to *SLCO1B1* genotype are presented in Figure 3.

**Figure 4:**
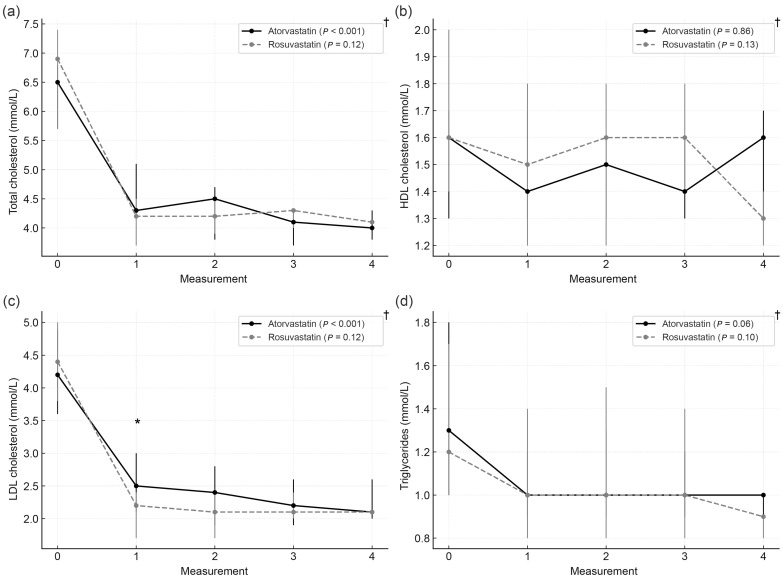
Lipid parameters by statin type within the *SLCO1B1* T/C genotype. (**a**) Total cholesterol, (**b**) HDL cholesterol, (**c**) LDL cholesterol, and (**d**) triglycerides during follow-up. Data are presented as medians with interquartile ranges. Measurement 0: baseline visit; 1–4: follow-up visits. * Mann–Whitney test *p* < 0.05; † Friedman test.

Within the *SLCO1B1* T/C genotype, participants treated with rosuvastatin achieved significantly lower LDL-C levels at the first follow-up compared with those receiving atorvastatin (median 2.2 [1.7–2.4] vs. 2.5 [2.1–3.0] mmol/L; *p* = 0.02). Other lipid parameters did not differ significantly between statin types. In the atorvastatin subgroup, total and LDL cholesterol levels showed a significant decline during follow-up, whereas in the rosuvastatin subgroup, lipid parameters remained stable after the initial reduction observed at the first visit. Median values by statin type within the T/C genotype are presented in Figure 4.

**Figure 5:**
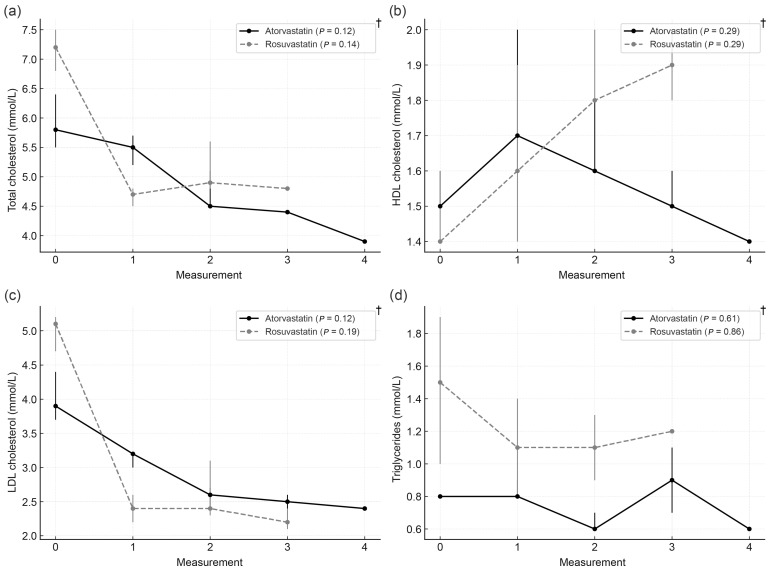
Lipid parameters by statin type within the *SLCO1B1* C/C genotype. (**a**) Total cholesterol, (**b**) HDL cholesterol, (**c**) LDL cholesterol, and (**d**) triglycerides during follow-up. Data are presented as medians with interquartile ranges. Measurement 0: baseline visit; 1–4: follow-up visits. † Friedman test (performed for measurements 0–3, as less than two participants was included in measurement 4.

Among participants with the *SLCO1B1* C/C genotype, no significant differences in lipid concentrations were observed between atorvastatin and rosuvastatin at any time point. Likewise, neither treatment produced meaningful changes in total cholesterol, HDL-C, LDL-C, or triglyceride levels during follow-up (Figure 5).

(c) According to baseline vitamin D concentrations

The distribution of baseline vitamin D categories (optimal, insufficient, deficient, and severely deficient) was similar between the atorvastatin and rosuvastatin groups, with no statistically significant differences (Table 4).

In the overall study population, baseline vitamin D concentration was not associated with the achievement of LDL-C targets (Table 5).

Serum vitamin D concentrations were subsequently grouped into two categories: optimal/insufficient/deficient (>10 ng/mL) and severe deficiency (≤10 ng/mL). This classification enabled a more precise assessment of lipid changes across the treatment groups. Among patients with severe vitamin D deficiency, no significant reduction in LDL levels was observed during follow-up, both in the atorvastatin group (Friedman test, *p* = 0.14) and in the rosuvastatin group (Friedman test, *p* = 0.12) (Table 6).

### 3.6. Predictors of LDL-C Target Achievement

In univariable logistic regression analysis, multiple clinical and lifestyle factors were evaluated as potential predictors of achieving the predefined LDL-C target, including age, age at menopause, duration of menopause, body mass index, waist-to-hip ratio, smoking status, alcohol consumption, daily physical activity, daily sedentary time, cardiovascular risk category, baseline vitamin D concentration, statin type, and SLCO1B1 genotype. Older age showed a trend toward a lower likelihood of LDL-C target attainment but did not reach statistical significance (OR 0.94, *p* = 0.09). In contrast, older age at menopause was significantly associated with reduced odds of achieving target LDL-C (OR 0.81, *p* = 0.008). Among lifestyle factors, daily alcohol consumption was associated with an 89% lower likelihood of LDL-C target attainment (OR 0.11, *p* = 0.02), while greater daily sedentary time was also a significant negative predictor (OR 0.99 per minute, *p* = 0.02). High cardiovascular risk category further reduced the probability of achieving LDL-C targets (OR 0.17, *p* = 0.009). Treatment with rosuvastatin significantly increased the likelihood of achieving target LDL-C levels (OR 2.81, *p* = 0.03). Baseline vitamin D concentration, smoking status, and SLCO1B1 genotype were not significant predictors of LDL-C target attainment in univariable analysis. Variables showing potential associations in univariable analysis, along with clinically relevant covariates, were subsequently included in a multivariate logistic regression model.

Multivariate logistic regression analysis was performed to identify independent predictors of achieving LDL-C targets in the present study. Variables entered into the model included age, age at menopause, duration of menopause, BMI, physical activity, cardiovascular (CV) risk, *SLCO1B1* genotype, type of statin, and baseline vitamin D concentration.

After stepwise selection, the final model retained age at menopause, daily passive activity, baseline cardiovascular risk category, and statin type as significant contributors (*p* < 0.001; overall classification accuracy 85.1%). Each additional year of age at menopause was associated with a 15% lower likelihood of achieving target LDL-C (OR 0.85, *p* = 0.060). Greater daily passive activity and baseline cardiovascular risk category were associated with a lower rate of LDL-C target attainment during follow-up. Participants classified as being at very high cardiovascular risk at baseline showed an approximately 85% lower rate of reaching LDL-C targets compared with those at moderate risk. Treatment with rosuvastatin independently increased the likelihood of LDL-C target attainment more than threefold compared with atorvastatin (Table 7).

### 3.7. Statin-Related Adverse Events

#### 3.7.1. Effect of Statin Type and Dose

Adverse effects related to statin therapy occurred in 15 participants (10.3%). Events were significantly more frequent in the atorvastatin group compared with the rosuvastatin group (18.3% vs. 2.7%; *p* = 0.002). Participants who started on high-dose therapy had a higher incidence of adverse events than those on moderate doses (14.3% vs. 2.1%; *p* = 0.02). Both cases in the rosuvastatin group occurred among women initiated on high-intensity treatment.

The proportion of participants with adverse events increased progressively across cardiovascular risk categories (2.4% in moderate, 9.3% in high, and 18.4% in very high risk; *p* for trend = 0.01). Among all affected participants, mild and transient elevations of liver transaminases (<3× upper limit of normal) were observed in two women receiving high-intensity therapy; both normalized spontaneously without discontinuation.

Gastrointestinal complaints were the most common: nausea (n = 6), bloating (n = 5), and mild right upper quadrant pain (n = 2). Most events (60%) occurred eight weeks after statin initiation. According to the Naranjo probability scale, most reactions were classified as “possible,” while two hepatic cases were “probable.” No participant experienced statin-associated muscle symptoms, creatine kinase elevation, or treatment discontinuation.

#### 3.7.2. Effect of SLCO1B1 c.521T>C Genotype

Among participants who experienced adverse events, 4 (26.6%) carried the T/T genotype, 10 (66.7%) the T/C genotype, and 1 (6.7%) the C/C genotype. Within genotype groups, adverse events occurred in 4.4% of T/T, 21.3% of T/C, and 14.3% of C/C carriers. A statistically significant association was observed between SLCO1B1 genotype and the occurrence of adverse events (Pearson χ^2^ = 9.646, df = 2, *p* = 0.008). Post hoc analysis with Bonferroni correction confirmed a significantly higher incidence among T/C heterozygotes compared with T/T homozygotes (Pearson χ^2^ = 9.689, df = 1, *p* = 0.002).

After stratification by treatment, the association between *SLCO1B1* genotype and adverse events remained significant in the atorvastatin group (Pearson χ^2^ = 17.286, df = 2, *p* < 0.001), whereas no significant association was observed in the rosuvastatin group (Pearson χ^2^ = 1.325, df = 2, *p* = 0.52), consistent with the lower number of adverse events in this group.

#### 3.7.3. Effect of Baseline Vitamin D Concentration

There was no statistically significant difference in baseline vitamin D concentrations between participants who experienced adverse events and those who did not (median 16.2 [14.2–22.0] vs. 21.1 [15.0–27.5] nmol/L; Mann–Whitney test, *p* = 0.37). Adverse events occurred in 3 participants (10.3%) with optimal baseline vitamin D levels, 2 (4.5%) with vitamin D deficiency, and 10 (16.4%) with insufficiency, while no adverse events were observed among participants with severe vitamin D deficiency. Overall, no statistically significant association was found between baseline vitamin D categories and the occurrence of adverse events (Pearson χ^2^ = 5.27, df = 3, *p* = 0.15).

#### 3.7.4. Predictors of Statin-Related Adverse Events

Multivariate logistic regression was conducted to identify independent predictors of adverse events. Variables entered into the model included age, age at menopause, cardiovascular risk category, baseline vitamin D concentration, *SLCO1B1* genotype, and statin type.

In the final model, atorvastatin therapy remained a significant independent predictor, increasing the likelihood of adverse events more than tenfold (OR 10.14, *p* = 0.004). The *SLCO1B1* T/C genotype was also independently associated with higher risk, conferring a sevenfold increase compared with the T/T genotype (OR 7.4, *p* = 0.002). The overall model was statistically significant (*p* < 0.001) and correctly classified 89.7% of cases (Table 8).

## 4. Discussion

### 4.1. Summary of Key Findings

This study examined how *SLCO1B1* c.521T>C polymorphism and serum vitamin D levels affect lipid response and tolerability to atorvastatin and rosuvastatin in postmenopausal women. Rosuvastatin demonstrated superior efficacy in achieving LDL-C targets, especially in women at higher cardiovascular risk. The *SLCO1B1* variant did not influence lipid-lowering response but was strongly associated with adverse effects, particularly among T/C carriers receiving atorvastatin. Vitamin D status showed no significant impact on lipid outcomes or adverse events; however, lower concentrations were linked to a higher frequency of mild side effects, suggesting a potential—though not statistically confirmed—role in treatment tolerance.

### 4.2. Pharmacokinetic Basis of Rosuvastatin Superiority

Our cohort of postmenopausal women demonstrated a substantial LDL-C reduction following statin initiation, confirming the efficacy of intensive statin therapy in this population. Rosuvastatin produced a faster and more pronounced LDL-C decline than atorvastatin, consistent with findings from large-scale comparative trials (STELLAR, URANUS) showing superior LDL-C goal attainment with rosuvastatin [19,20]. Despite similar baseline characteristics, rosuvastatin users more frequently reached therapeutic targets, and triglyceride reduction was also more marked in this group, while HDL-C changes remained modest across both treatments. The overall LDL-C goal attainment rate of 74.5% exceeded most European real-world reports (≈50%), likely reflecting the consistent use of high-intensity statins and structured follow-up [21].

The greater lipid-lowering efficacy of rosuvastatin can be attributed to its pharmacokinetic and pharmacodynamic profile. Compared with atorvastatin, rosuvastatin exhibits stronger binding affinity for HMG-CoA reductase, greater hepatoselectivity, and a longer elimination half-life, producing more sustained inhibition of hepatic cholesterol synthesis [17,22]. Owing to minimal first-pass metabolism—less than 10% of the administered dose is converted into inactive metabolites—rosuvastatin achieves higher bioavailability (~20% vs. 14% for atorvastatin), resulting in greater intrahepatic exposure and enhanced LDL-C lowering [22,23]. Its hydrophilic nature further limits extrahepatic diffusion, favoring selective uptake into hepatocytes via organic anion transporter (OATP) family members 1B1, 2B1, 1B3, 1A2 and sodium-dependent taurocholate cotransporting polypeptide (NTCP) which may also contribute to the lower incidence of systemic adverse effects [24].

The predominance of high-intensity therapy in both groups (approximately two-thirds of participants) also contributed to the overall treatment success, in line with major outcome trials demonstrating that greater statin intensity yields superior cardiovascular protection. In the TNT trial, atorvastatin 80 mg reduced LDL-C to 2.0 mmol/L and lowered major cardiovascular events by 22% compared with 10 mg daily [25], while in JUPITER, rosuvastatin 20 mg reduced LDL-C by about 50% and high-sensitivity C-reactive protein (hs-CRP) by 37%, leading to significant event reduction without an increased risk of myopathy [26]. The PROVE-IT TIMI-22 study similarly confirmed early benefits of intensive LDL-C lowering after acute coronary syndrome [27]. Even moderate-intensity therapy has proven more effective than non-pharmacological alternatives—in the SPORT trial, rosuvastatin 5 mg/day reduced LDL-C by ~35% within four weeks, whereas none of six common dietary supplements produced meaningful changes [28].

### 4.3. Comparison with Real-World Data

Compared with contemporary European real-world data, the LDL-C target attainment rate in our cohort was substantially higher. In the cross-sectional DA VINCI study, which included patients from 18 European countries, only 54% achieved LDL-C goals according to the 2016 ESC/EAS guidelines, and merely 33% met the stricter 2019 targets, with attainment particularly low among very-high-risk individuals [21]. The limited use of high-intensity statins (20% in primary and 38% in secondary prevention) and the infrequent application of combination therapy (statin plus ezetimibe 9%, PCSK9 inhibitors 1%) largely explained these suboptimal outcomes. More recent evidence from the SANTORINI study, which enrolled over 9000 patients across 14 European countries, confirmed that these therapeutic gaps persist despite guideline updates. At baseline, only 20% of high- and very-high-risk patients achieved LDL-C goals recommended by the 2019 ESC/EAS guidelines, while 80% remained above target despite broad awareness and availability of lipid-lowering therapies. Notably, 21.8% of patients were not receiving any lipid-lowering treatment, and more than half were on statin monotherapy, emphasizing the continued underutilization of combination approaches and treatment intensification [29].

In contrast, the markedly higher goal-attainment rate observed in our study (74.5%) likely reflects the systematic use of potent, high-intensity statins from the outset and proactive, closely monitored dose adjustments throughout follow-up. Such an individualized and well-supervised therapeutic strategy minimizes clinical inertia, facilitates timely dose optimization, and maximizes lipid-lowering efficacy.

### 4.4. Impact of SLCO1B1 Polymorphism on Statin Response

The genotype distribution of *SLCO1B1* c.521T>C (p.Val174Ala; rs4149056) in our cohort (T/T 62.8%, T/C 32.4%, C/C 4.8%) corresponded to a C-allele frequency of 21%, closely matching previously reported values in the Croatian population (T/T 61.7%, T/C 34.8%, C/C 3.5%; C allele ≈ 20.9%) [30], confirming the representativeness of our sample.

Among T/T carriers, both atorvastatin and rosuvastatin achieved comparable reductions in total and LDL cholesterol, reflecting preserved OATP1B1 transporter activity and indicating that between-statin differences in this group were pharmacologic rather than pharmacogenetic.

In contrast, T/C heterozygotes exhibited a more rapid LDL-C decline with rosuvastatin, while atorvastatin produced a gradual, dose-dependent response over follow-up—likely reflecting its stronger reliance on OATP1B1-mediated hepatic uptake [7,9]. These observations align with rosuvastatin’s partial independence from OATP1B1 transport and its additional reliance on other hepatic transporters, including OATP1B3 and OATP2B1, as well as ABCG2/BCRP for biliary excretion [7].

In the small C/C subgroup, lipid responses were attenuated for both statins, consistent with prior reports showing higher on-treatment LDL-C levels among carriers of the C allele. The C/C genotype is associated with reduced hepatic uptake and increased systemic exposure, with reported AUC increases of approximately 40–81% for atorvastatin and 19–68% for rosuvastatin [7,31]. However, since HMG-CoA reductase inhibition occurs within hepatocytes, elevated plasma concentrations do not necessarily translate to greater lipid-lowering efficacy [9,32]. Consequently, *SLCO1B1* variants predominantly affect statin tolerability rather than lipid-lowering potency, as confirmed by meta-analyses showing no consistent association between the c.521T>C polymorphism and LDL-C reduction [33].

The pharmacogenetic mechanisms underlying these patterns are rooted in the metabolic and transport characteristics of each statin. Atorvastatin is primarily metabolized in the liver via the cytochrome P450 system, mainly CYP3A4 and CYP3A5, creating a substantial potential for drug–drug interactions, particularly with CYP3A4 inhibitors [34]. It undergoes extensive hepatic metabolism to active ortho- and parahydroxylated metabolites and various β-oxidation products, while also being subject to glucuronidation mediated by UGT1A1, UGT1A3, and UGT2B7 [23,35]. Atorvastatin’s hepatic uptake depends mainly on OATP1B1, encoded by *SLCO1B1*, whereas efflux transporters modulate its intestinal absorption and biliary excretion [36,37].

Rosuvastatin undergoes limited metabolism, with CYP2C9 as the principal enzyme involved [17,38]. Its pharmacokinetics are influenced by both SLCO1B1 and ABCG2 polymorphisms, with the SLCO1B1 exerting a smaller effect than observed for atorvastatin due to rosuvastatin’s higher hydrophilicity and broader transporter profile, including OATP1B3, OATP2B1, and OATP1A2. The ABCG2 c.421C>A polymorphism (rs2231142, p.Gln141Lys) further modulates systemic exposure, with homozygous 421A/A carriers exhibiting up to 144% higher AUC compared with wild-type [7,17].

Together, these pharmacogenetic insights explain the modest impact of *SLCO1B1* polymorphism on LDL-C lowering but its strong association with statin-related adverse events, particularly for atorvastatin, whose hepatic uptake relies almost exclusively on OATP1B1 transport.

### 4.5. Role of Vitamin D in Statin Efficacy and Tolerability

The mean serum vitamin D concentration in our cohort was 20 ng/mL (≈ 50 nmol/L), indicating widespread insufficiency among postmenopausal women. Comparable results were reported in a previous Croatian study, where 92.5% of women had levels below 30 nmol/L [39], consistent with the high prevalence of hypovitaminosis D observed across Europe, affecting more than 40% of adults and up to 85% of older individuals [40,41]. This deficiency largely reflects reduced cutaneous synthesis, limited sunlight exposure, and hormonal changes that accompany menopause.

The role of vitamin D in lipid metabolism remains controversial, with studies yielding inconsistent results. In our study, baseline vitamin D concentrations were not associated with differences in LDL-C target attainment. A large cross-sectional study including over 15,000 participants reported an inverse association between serum vitamin D and LDL-C levels, while no clear relationship was found with total cholesterol, HDL-C, or triglycerides [42]. Conversely, a recent meta-analysis of 25 studies demonstrated that vitamin D supplementation modestly reduced triglycerides and total cholesterol and increased HDL-C, whereas the effect on LDL-C was generally weak and inconsistent [43].

Vitamin D plays an important role in lipid metabolism by influencing bile acid synthesis and the expression of enzymes involved in cholesterol homeostasis, including HMG-CoA reductase [11,12]. Vitamin D deficiency may therefore contribute to enhanced endogenous cholesterol synthesis. In addition, vitamin D has been shown to indirectly affect lipid homeostasis by modulating triglyceride synthesis, parathyroid hormone activity, and insulin sensitivity, thereby contributing to dyslipidemia and atherogenesis [11,44,45].

Although baseline vitamin D concentrations were not significantly associated with LDL-C goal attainment, women with severe deficiency failed to achieve a meaningful LDL-C reduction despite high-intensity statin therapy. Similar findings have been reported previously, where atorvastatin effectively lowered LDL-C only in patients with vitamin D > 30 nmol/L [46]. This suggests that severe vitamin D deficiency may blunt the hepatic response to statins. thereby limiting statin uptake into hepatocytes—the primary site of action [47]. This mechanism is supported by experimental in vitro findings: in a study conducted on the human hepatocyte-derived Huh7 cell line, vitamin D deficiency reduced the expression of OATPs, suggesting a possible limitation of statin uptake [47].

However, these findings remain largely experimental, and direct effects of vitamin D on transporter genes *SLCO1B1* and ABCG2 expression in humans have not been confirmed. Clinically, our results imply that assessing and correcting vitamin D deficiency could be valuable in patients who do not reach LDL-C targets despite adequate adherence and therapy intensity. In postmenopausal women, optimizing vitamin D status may therefore enhance both cardiometabolic and skeletal health and should be considered part of comprehensive risk management.

### 4.6. Impact of Alcohol Consumption on LDL-C Target Attainment and Statin Pharmacokinetics

In our study, daily alcohol consumption was associated with an 89% lower likelihood of achieving LDL-C targets. Alcohol exerts dose-dependent effects on cardiometabolic health: while low intake may have modest protective effects, higher consumption is linked to metabolic syndrome components, including abdominal obesity, hypertriglyceridemia, and hyperglycemia, despite favorable HDL changes [48,49]. Women are more susceptible to alcohol-related harm due to biological differences, a vulnerability that is further accentuated during the peri- and postmenopausal period, when the potential benefits of moderate consumption are outweighed by the risks of chronic or excessive use [50].

In addition, alcohol consumption can significantly influence statin pharmacokinetics and pharmacodynamics through modulation of hepatic cytochrome P450 enzymes, particularly CYP2E1 and, to a lesser extent, CYP1A2. Chronic alcohol intake induces CYP2E1 activity, increasing hepatic oxidative stress and altering drug metabolism, which may enhance the biotransformation of statins metabolized via CYP pathways and increase susceptibility to statin-related hepatotoxicity or myopathy through the generation of reactive oxygen species and toxic intermediates [51,52].

### 4.7. Adverse Events

#### 4.7.1. Influence of Statin Type and Dose

In the total cohort, statin-related adverse events were reported in 15 participants (10.3%), occurring significantly more often in the atorvastatin group than in the rosuvastatin group. This difference aligns with the pharmacologic properties of lipophilic statins, which more readily penetrate extrahepatic tissues, including skeletal muscle, and are therefore linked with a higher risk of systemic adverse effects. Conversely, the hydrophilic profile of rosuvastatin limits extrahepatic diffusion and reduces the likelihood of SAMS [53].

Recent meta-analyses have reported an overall incidence of 4–5% for statin-related adverse effects, with higher rates observed in older adults and women. Chronic comorbidities such as diabetes, hypothyroidism, and obesity have been positively associated with adverse events, whereas hypertension has not been identified as a contributing factor [54]. Mild, transient elevations in liver enzymes are more commonly seen with higher doses of atorvastatin (40 mg/day) than with rosuvastatin (20 mg/day) [55].

Both statins have been associated with new-onset diabetes, although statistical significance has been consistently confirmed only in the SPARCL and JUPITER trials [56]. Some analyses have suggested a slightly greater risk of new-onset diabetes with rosuvastatin compared with atorvastatin when LDL-C levels fall below 1.8 mmol/L [57].

In our study, adverse events were more frequent among participants receiving high-intensity therapy at baseline, consistent with the known dose-dependent relationship between statin exposure and adverse events [58,59]. The progressive increase in adverse event rates across cardiovascular risk categories—from 2.4% in moderate-risk to 18.4% in very-high-risk participants—can be attributed to more aggressive treatment targets and polypharmacy in higher-risk individuals, both of which may increase susceptibility to drug interactions and side effects.

None of our participants developed muscle-related symptoms. Reported adverse events were mild and transient gastrointestinal disturbances (nausea, bloating, abdominal discomfort) or moderate, reversible increases in transaminases (ALT, AST < 3× ULN). These findings are in line with previous trials reporting constipation and abdominal discomfort in 2–3% of statin users and clinically relevant transaminase elevations in fewer than 1% [60,61,62]. The absence of myalgia in our cohort may reflect the generally healthy status of participants—postmenopausal women without diabetes, hypothyroidism, or renal disease—and their largely sedentary lifestyle, which reduced the risk of muscle-related symptoms [63,64,65]. Furthermore, none of the women were taking CYP3A4-interacting medications such as verapamil, diltiazem, or certain diuretics, which can elevate systemic statin concentrations and potentiate myotoxicity [64]. Overall, these data confirm good tolerability of both statins in this population.

#### 4.7.2. Influence of *SLCO1B1* c.521TC Polymorphism

A significant association between *SLCO1B1* genotype and the occurrence of adverse events was observed, with the highest incidence among T/C heterozygotes. This relationship remained significant in the atorvastatin subgroup but not in the rosuvastatin subgroup, likely due to the smaller number of adverse events and pharmacokinetic differences between the two statins. Carriers of the C allele have reduced OATP1B1 transporter function, leading to higher systemic exposure and an increased risk of statin-induced myopathy or hepatotoxicity [66].

Although our cohort reported only mild gastrointestinal adverse effects and no SAMS, these results reinforce previous findings that the *SLCO1B1* c.521T>C polymorphism is a major genetic determinant of statin intolerance, particularly for statins such as simvastatin and atorvastatin that rely heavily on OATP1B1-mediated hepatic uptake [67]. Clinical studies have shown cumulative myopathy rates as high as 18% in CC homozygotes treated with high-dose simvastatin, compared with only 0.6% in TT carriers, with over 60% of cases attributable to the C allele [66].

#### 4.7.3. Vitamin D and Adverse Events

Given vitamin D’s role in muscular and mitochondrial function, deficiency has been proposed as a potential risk factor for statin intolerance through impaired calcium homeostasis, decreased mitochondrial enzyme activity, and altered membrane integrity. Some studies have demonstrated a link between low vitamin D levels and higher rates of SAMS, although results remain inconsistent [68].

In our study, no association between vitamin D status and adverse events was found, likely due to the predominance of gastrointestinal rather than muscular symptoms. It is also possible that participants with lower vitamin D levels were prescribed lower statin doses, attenuating any potential adverse effect. The limited number of participants in the severe deficiency subgroup further reduced statistical power. Nevertheless, the biological plausibility of vitamin D’s influence on statin tolerance warrants confirmation in larger and longer-term studies.

#### 4.7.4. Effect of Statins on Hepatic Enzymes and CK

No significant differences in liver enzyme changes were observed between moderate- and high-intensity statin users, although transient ALT and AST elevations were more frequent with higher doses. This dose-dependent effect likely reflects a reversible adaptive hepatic response rather than true hepatotoxicity [69]. Literature data show that clinically significant (>3× ULN) transaminase elevations occur in <1% of patients and usually resolve without treatment interruption [70,71].

In our cohort, no elevations in creatine kinase or cases of myalgia, myopathy, or rhabdomyolysis were recorded, consistent with randomized trial data showing that clinically significant statin-induced myopathy occurs in fewer than 0.1% of patients on maximal doses [69]. The absence of muscle toxicity in our participants—who were generally healthy and not exposed to drug interactions—further confirms that atorvastatin and rosuvastatin are well tolerated in postmenopausal women.

### 4.8. Clinical Implications and Future Directions

This study has several notable strengths. It included a well-defined cohort of postmenopausal women with uniform inclusion criteria and standardized lipid monitoring across all visits. The prospective design with monthly biochemical follow-up enabled accurate tracking of lipid and safety parameters, while specialist supervision ensured consistent treatment intensity and high adherence. Moreover, by combining vitamin D assessment with *SLCO1B1* genotyping, this research provides a translational link between endocrine and pharmacogenetic determinants of statin response—an approach rarely explored in similar populations.

However, several limitations should be acknowledged. The study included a moderate number of participants recruited from a single geographic region and comprised exclusively postmenopausal women of European ancestry, which may limit the generalizability of the findings to other ethnic populations and to men. Sex-specific differences in statin pharmacokinetics and pharmacodynamics, as well as ethnic variability in allele frequencies and genetic backgrounds, should therefore be considered when interpreting the results.

The 16-week follow-up period allowed assessment of short-term lipid-lowering efficacy and tolerability but was not designed to evaluate long-term cardiovascular outcomes or sustained safety profiles.

In addition, outcome assessors were not blinded, which may have introduced assessment bias, particularly in the evaluation of subjective adverse events. However, the primary efficacy and safety outcomes were based on objective laboratory measurements, which reduces the likelihood of significant systematic bias.

Furthermore, pharmacogenetic analysis was restricted to the *SLCO1B1* c.521T>C polymorphism. While this variant represents the most extensively studied determinant of statin transport and myopathy risk, the absence of other relevant transporter (e.g., ABCG2) and metabolizing enzyme gene analyses may have limited the ability to capture the full genetic contribution to interindividual variability in statin response. In addition, the low frequency of participants carrying the CC genotype of *SLCO1B1* represents an important limitation, as the study may have been underpowered to detect small-to-moderate genotype effects on lipid response within this genotype.

Although treatment adherence was high, residual confounding related to dietary habits, physical activity, or sun exposure affecting vitamin D status cannot be fully excluded.

Future studies should include larger and more diverse cohorts with extended follow-up to confirm these findings and assess their impact on clinical outcomes. Expanding pharmacogenetic profiling to include *ABCG2* and *CYP* variants, as well as integrating pharmacokinetic modeling, could help refine individualized statin dosing strategies. Interventional research on optimizing vitamin D status in parallel with pharmacogenetic analysis may further clarify mechanisms influencing efficacy and tolerability in women.

## 5. Conclusions

In Croatian postmenopausal women, rosuvastatin demonstrated greater LDL-C–lowering efficacy and better tolerability than atorvastatin, independent of *SLCO1B1* genotype. The *SLCO1B1* c.521T>C polymorphism was not associated with lipid response but significantly influenced the occurrence of adverse effects, particularly among atorvastatin-treated carriers of the T/C genotype. Vitamin D deficiency did not significantly affect lipid outcomes or safety, although severely deficient individuals showed a trend toward poorer statin response. These findings highlight the importance of integrating pharmacogenetic and endocrine parameters into individualized lipid-lowering strategies, especially in women at higher cardiovascular risk. Future studies with larger and more diverse populations and longer follow-up periods are warranted to confirm these associations and explore their impact on long-term cardiovascular outcomes.

## Figures and Tables

**Table 1:** General characteristics of all participants.

Variable	Median (IQR) or *N* (%) (*N* = 145)
Age (years)	60 (55–64)
Age at menopause (years)	50 (48–52)
Duration of menopause (years)	9 (5–14)
Body weight (kg)	74 (67–83)
Height (cm)	164 (161–169)
Body mass index (kg/m^2^)	26.99 (24.06–30.86)
Waist circumference (cm)	88 (80.5–99)
Hip circumference (cm)	106 (100–113)
Waist-to-hip ratio	0.84 (0.79–0.89)
Employment status	
• Employed	86 (59.3)
• Unemployed	5 (3.4)
• Retired	54 (37.2)
Education level	
• Primary school	3 (2.1)
• High school	97 (66.9)
• Associate degree	14 (9.7)
• University degree	26 (17.9)
• Professional specialist	3 (2.1)
• Doctorate	2 (1.4)
Smoking status	
• Non-smoker	72 (49.7)
• Former smoker	24 (16.6)
• Occasional smoker	5 (3.4)
• Regular smoker	44 (30.3)
Alcohol consumption (past year)	
• No	71 (49.7)
• Daily	5 (3.4)
• Weekly	8 (5.5)
• Monthly	13 (9.0)
• Several times per year	47 (32.4)
Physical activity	
• Moderate/vigorous activity per day (min)	120 (75–200)
• Sedentary time per day (min)	240 (120–360)

Categorical variables are presented as absolute and relative frequencies; continuous variables are presented as median (interquartile range).

**Table 2:** Statin dose distribution during follow-up according to statin type.

Visit	Dose Category	Atorvastatin *n* (%)	Rosuvastatin *n* (%)	*p* *
Baseline	Moderate dose	24 (33.8)	23 (31.1)	0.73
	High dose	47 (66.2)	51 (68.9)	
1st follow-up	Moderate dose	14 (26.4)	11 (20.8)	0.49
	High dose	39 (73.6)	42 (79.2)	
2nd follow-up	Moderate dose	0	2 (6.1)	0.17
	High dose	30 (100)	31 (93.9)	
3rd follow-up	Moderate dose	0	0	–
	High dose	18 (100)	10 (100)	

* Pearson χ^2^ test.

**Table 3:** LDL-C Target Attainment by Statin Type.

Statin Group	Achieved LDL-C Target, *n* (%)	Did not Achieve LDL-C Target, *n* (%)	Total (*n*)	*p* *
Atorvastatin	48 (67.6)	23 (32.4)	71	
Rosuvastatin	60 (81.1)	14 (18.9)	74	0.02

Legend: LDL-C targets were defined according to the 2021 ESC/EAS dyslipidemia guidelines. * Pearson χ^2^ test.

**Table 4:** Distribution of vitamin D level categories according to statin type.

Vitamin D Concentration Category	Atorvastatin *n* (%)	Rosuvastatin *n* (%)	*p* *
Optimal (≥30 ng/mL)	16 (22.5)	13 (17.6)	0.45
Insufficiency (20–29.9 ng/mL)	21 (29.6)	23 (31.1)	
Deficiency (10–19.9 ng/mL)	31 (43.7)	30 (40.5)	
Severe deficiency (<10 ng/mL)	3 (4.2)	8 (10.8)	

* Pearson χ^2^ test.

**Table 5:** Frequency of LDL-C target achievement according to baseline vitamin D category.

Follow-Up Visit	Optimal Vitamin D *N* (%)	Deficient Vitamin D *N* (%)	Insufficient Vitamin D *N* (%)	Severe Vitamin D Deficiency *N* (%)	*p* *
LDL-C target achieved					
1st follow-up	9/29 (31.0)	15/44 (34.1)	12/61 (19.7)	2/11 (18.2)	0.34
2nd follow-up	9/19 (47.4)	11/28 (39.3)	18/49 (36.7)	5/9 (55.6)	0.68
3rd follow-up	4/10 (40.0)	5/17 (29.4)	12/31 (38.7)	3/4 (75.0)	0.42
4th follow-up	0/6 (0.0)	2/8 (25.0)	1/14 (7.1)	0/1 (0.0)	0.42

Legend: Data are presented as number of patients achieving LDL-C targets/total number in category (%). * Pearson χ^2^ test.

**Table 6:** Longitudinal LDL-C Changes According to Baseline Vitamin D Status in Atorvastatin and Rosuvastatin Groups.

Statin Group	Vitamin D Category	Baseline LDL-C Median (IQR), mmol/L	Final Follow-Up LDL-C Median (IQR), mmol/L	*p* †
Atorvastatin	Optimal/mild deficiency	4.2 (3.7–4.7)	2.2 (1.9–2.5)	<0.001
Atorvastatin	Severe deficiency	3.6 (3.2–4.2)	2.0 (1.8–2.2)	0.14
Rosuvastatin	Optimal/mild deficiency	4.4 (3.8–5.0)	2.1 (2.0–2.4)	<0.001
Rosuvastatin	Severe deficiency	4.3 (3.6–5.0)	2.1 (2.1–2.1)	0.12

Legend: Data are presented as median (interquartile range). † *p* values represent within-group longitudinal changes assessed using the Friedman test.

**Table 7:** Multivariate logistic regression analysis—predictors of achieving target LDL-C levels.

Predictor	β	Wald	*p*	OR	95% CI
Age at menopause (years)	–0.158	3.665	0.060	0.85	0.73–1.00
Daily passive activity (min)	–0.005	7.245	0.007	0.99	0.99–1.00
CV risk					
• High	–1.622	4.516	0.030	0.20	0.04–0.88
• Very high	–1.900	6.521	0.010	0.15	0.03–0.64
Statin type (Rosuvastatin)	1.148	4.873	0.030	3.15	1.14–8.73
Constant	11.590	7.477	0.006	—	—

Legend: β—regression coefficient; OR—odds ratio; CI—confidence interval.

**Table 8:** Multivariate logistic regression analysis—predictors of statin-related adverse events.

Predictor	β	Wald	*p*	OR	95% CI
Statin type (Atorvastatin)	2.314	8.290	0.004	10.1	2.1–48.9
SLCO1B1 c.521T>C (ref. *T/T*)					
• T/C	2.002	9.355	0.002	7.4	2.1–26.7
• C/C	1.531	1.463	0.23	4.62	0.39–55.2
Constant	–4.773	28.780	<0.001	—	—

Legend: β—regression coefficient; OR—odds ratio; CI—confidence interval.

## Data Availability

The original contributions presented in the study are included in the article, further inquiries can be directed to the corresponding author/s, T.B., upon reasonable request.

## Abbreviations

The following abbreviations are used in this manuscript:

25(OH)D3	25-hydroxyvitamin D3
ABCG2	ATP-binding cassette subfamily G member 2
ALT	Alanine aminotransferase
AST	Aspartate aminotransferase
AUC	Area under the curve
BMI	Body mass index
CK	Creatine kinase
CV	Cardiovascular
CVD	Cardiovascular disease
ESC/EAS	European Society of Cardiology / European Atherosclerosis Society
GGT	Gamma-glutamyl transferase
HDL-C	High-density lipoprotein cholesterol
HMG-CoA	3-hydroxy-3-methylglutaryl–coenzyme A
IQR	Interquartile range
LC–MS/MS	Liquid chromatography–tandem mass spectrometry
LDL-C	Low-density lipoprotein cholesterol
NTCP	Sodium-dependent taurocholate cotransporting polypeptide
OATP	Organic anion transporting polypeptide
OR	Odds ratio
PCR	Polymerase chain reaction
PCSK9	Proprotein convertase subtilisin/kexin type 9
SAMS	Statin-associated muscle symptoms
SD	Standard deviation
SLCO1B1	Solute Carrier Organic Anion Transporter Family Member 1B1
SPSS	Statistical Package for the Social Sciences
TC	Total cholesterol
TG	Triglycerides
ULN	Upper limit of normal
VDR	Vitamin D receptor
